# Sir James Crichton Browne's Cure for Quackery

**Published:** 1894-10-27

**Authors:** 


### The Hospital

AN INSTITUTIONAL JOURNAL OF

The Medical Sciences and Hospital Administration,

WITH

VOL. XVII.—No. 422.] AN EXTRA NURSING SUPPLEMENT. [OCT. 27, 1894.

### SIR JAMES CRICHTON BROWNE'S CURE FOR QUACKERY.

A LECTURE to working men on "Tuberculosis" by a man of high and undoubted standing in the medical profession is a novelty. Such a lecture was delivered by Sir James Crichton Browne in connection with the Congress of the Sanitary Institute at Liverpool the other day. Concerning the lecture itself, the *Times* in a critical and appreciative leader declared that it was "as interesting as a romance, and as accurate as a professorial address." The subject, it must be admitted, was one of the very profoundest interest to all human beings, and if in times past it has been held to be caviare to the general, it has been because knowledge has been lacking and speculation has been crude and incompetent.

The novelty of Sir James Crichton Browne's lecture does not consist in the newness of the theories broached or the facts announced, for they are not new as we now count scientific things new, at any rate to medical men. It consists in the fact that such an address should have been prepared for working men, and should have been listened to by them, not only without weariness but with interest increasing to the end. It is true Sir James Crichton Browne is an admirable expositor, and the lantern-slide is the most delightful and persuasive of instructors. But even admitting this we still feel that the old-fashioned practitioner would have anticipated a second deluge if he had been told that before the nineteenth century was out the working classes, instructed by the highest medical authorities, would know more about tuberculosis than he had been able to discover in a long lifetime.

Some of the facts announced to the audience must have struck the working men as exceedingly wonderful, and must have given them quite new ideas as to the marvellousness of the world in which they live and as to the extraordinary scientific skill of the men who devote themselves to the purely scientific life. When, for example, the lecturer related that as many as 400,000,000 microbes, pathogenic and otherwise, could all stand at the same moment on a single postage stamp, the listeners must have felt that there were many things in heaven and earth not dreamed of in the working man's philosophy. Similarly the brewing of beer, the fermenting of wine, the leavening of bread, the nitrification of the soil, the scavenging by means of the chemical changes set up in decomposing matter—all these everyday circumstances set forth in their full scientific significance in simple language and with the facts and processes themselves shown in all but actual operation on the illuminated screen, must have filled the audience with some of that consciousness of ignorance which is the parent of all new knowledge and of all worthy culture of the mind.

Many may be the results of such a lecture in the mind of every intelligent workman, and, indeed, of every thinking person both within and without the medical profession. It is impossible that facts, so striking in themselves, and charged with such significance for all the human family, can be brought into the consciousness of any intelligent human being, and leave no profitable fruit behind. The working man and all the laity are gradually learning to look on the medical profession with new eyes. Reflection will suggest to the least intelligent that the scientists whose arduous labours have discovered these facts, and not only discovered them but brought them out of the most secret recesses of the world's mysteries into the full light of day, so that now even the child may understand them, cannot be placed in the category of mere self-seekers and money-loving adventurers, but must be regarded as among the purest illuminators and benefactors of mankind. Similarly the whole class of medical men whose lives are spent in the daily application of such knowledge at the bedside, must be separated by an instructed public from the charlatan, the wicked charlatan, who, with no scientific knowledge whatever, takes up some probably harmless, but mostly quite useless substance, and with impudent assurance demands money on the strength of promises which he does not even understand, much less know how to perform.

The service which Sir James Crichton Browne and other such lecturers are rendering to medicine is this, that they are giving to the masses of the people the means of judging it fairly. In past times those means have not been furnished to the public; and we can partly understand why. In past times medicine was not scientific; it was speculative; evolved from the mind, and not gathered patiently by observation from nature and life. As a consequence it was sometimes foolish, often unpractical, and not seldom injurious. It need not be wondered at, therefore, that medical men recognised the safety of keeping their knowledge and speculations, such as they were, strictly to themselves. But, now, all this is changed, and changed absolutely. The past of medicine is, for all practical purposes, a closed book. Physic to-day is as resolutely scientific as it has been unscientific in past times. Every new discovery in physic passes through the crucible, or the hundreds of crucibles, of competing scientists before it is given to the outside world. A theory or a discovery which is alive at the termination of such a process has little to fear from the criticisms of those who are not medical.

For the last two or three decades the practitioner of medicine has been passing through a very searching crucible of his own. Brought up in the old, he has had, in maturity, to discard it in favour of the new. The new, too, has been so very new. It has shown medical men their ignorance with a convincing thoroughness which has left nothing to be desired. But whilst robbing them of an empirical assurance which often led to success in practice, it has filled them with doubts which have produced in some men actual paralysis of effort and hope. During this period of incertitude there has been an enormous development of quackery. If fools rush in where angels fear to tread, much more do knaves assume unwonted boldness when honesty, half-blinded by excess of light, walks cautiously in order that it may walk surely. But better days, we feel sure, are in store for scientific medical practice; and the way to hasten the advent of such days is to do as Sir James Crichton Browne has done, and to instruct our critics, the public, as widely and thoroughly as possible.

